# Genome-wide association study reveals ethnicity-specific SNPs associated with ankylosing spondylitis in the Taiwanese population

**DOI:** 10.1186/s12967-022-03701-3

**Published:** 2022-12-12

**Authors:** Ching-Lung Ko, Wei-Zhi Lin, Meng-Ting Lee, Yu-Tien Chang, Hung-Che Lin, Yi-Syuan Wu, Jun-Fu Lin, Ke-Ting Pan, Yu-Chuan Chang, Ko-Han Lee, Yi-Lun Lee, Tsung-Ting Hsieh, Jia-Hsin Huang, Chih-Hung Wang, Sung-Sen Yang, Hsiang-Cheng Chen, Chi-Ming Chu

**Affiliations:** 1 Graduate Institute of Life Sciences, National Defense Medical Center, Taipei, 114 Taiwan; 2 Department of Anesthesiology, Wan Fang Hospital, Taipei Medical University, Taipei, 116 Taiwan; 3 School of Public Health, National Defense Medical Center, Taipei, 114 Taiwan; 4 Department of Otolaryngology-Head and Neck Surgery, Tri-Service General Hospital, National Defense Medical Center, Taipei, 114 Taiwan; 5 Trauma and Critical Care Service, Department of Surgery, Kaohsiung Medical University Hospital, Kaohsiung Medical University, Kaohsiung, 807 Taiwan; 6 Graduate Institute of Aerospace and Undersea Medicine, National Defense Medical Center, Taipei, 114 Taiwan; 7 AI Labs, Taipei, 10351 Taiwan; 8 Graduate Institute of Medical Sciences, National Defense Medical Center, Taipei, 114 Taiwan; 9 Division of Nephrology, Department of Internal Medicine, Tri-Service General Hospital, National Defense Medical Center, Taipei, 114 Taiwan; 10 Division of Rheumatology/Immunology and Allergy, Department of Internal Medicine, Tri-Service General Hospital, National Defense Medical Center, Taipei, 114 Taiwan; 11 Big Data Research Center, College of Medicine, Fu-Jen Catholic University, New Taipei, 242 Taiwan; 12 Department of Public Health, Kaohsiung Medical University, Kaohsiung, 807 Taiwan; 13 Department of Public Health, China Medical University, Taichung, 406 Taiwan

**Keywords:** Genome-wide association study, Ankylosing spondylitis, Single-nucleotide polymorphism, Taiwanese, SNPs, HLA-B27

## Abstract

**Background:**

Ankylosing spondylitis (AS) is an autoimmune disease affecting mainly spine and sacroiliac joints and adjacent soft tissues. Genome-wide association studies (GWASs) are used to evaluate genetic associations and to predict genetic risk factors that determine the biological basis of disease susceptibility.

We aimed to explore the race-specific SNP susceptibility of AS in Taiwanese individuals and to investigate the association between HLA-B27 and AS susceptibility SNPs in Taiwan.

**Methods:**

Genotyping data were collected from a medical center participating in the Taiwan Precision Medicine Initiative (TPMI) in the northern district of Taiwan. We designed a case–control study to identify AS susceptibility SNPs through GWAS. We searched the genome browser to find the corresponding susceptibility genes and used the GTEx database to confirm the regulation of gene expression. A polygenic risk score approach was also applied to evaluate the genetic variants in the prediction of developing AS.

**Results:**

The results showed that the SNPs located on the sixth chromosome were related to higher susceptibility in the AS group. There was no overlap between our results and the susceptibility SNPs found in other races. The 12 tag SNPs located in the MHC region that were found through the linkage disequilibrium method had higher gene expression. Furthermore, Taiwanese people with HLA-B27 positivity had a higher proportion of minor alleles. This might be the reason that the AS prevalence is higher in Taiwan than in other countries. We developed AS polygenic risk score models with six different methods in which those with the top 10% polygenic risk had a fivefold increased risk of developing AS compared to the remaining group with low risk.

**Conclusion:**

A total of 147 SNPs in the Taiwanese population were found to be statistically significantly associated with AS on the sixth pair of chromosomes and did not overlap with previously published sites in the GWAS Catalog. Whether those genes mapped by AS-associated SNPs are involved in AS and what the pathogenic mechanism of the mapped genes is remain to be further studied.

**Supplementary Information:**

The online version contains supplementary material available at 10.1186/s12967-022-03701-3.

## Introduction

Ankylosing spondylitis (AS), an autoinflammatory disorder, is an unusual but well-known cause of chronic back pain. Common signs of AS are joint pain and stiffness, typically occurring at joints of the spine, as well as in the pelvis, shoulders, or hips. AS may progress to symptoms such as deformed joints, limited lumbar movement, and reduced thoracic vertebral activity. Endochondral ossification slowly progressing to fusions of spinal segments is a major cause of the symptoms. The extreme pattern can lead to the bony fusion of vertebral joints and eventually become a disability. Currently, AS is incurable and thought to be caused mainly by uncharacterized genetic factors [1].

The prevalence of AS per ten thousand is approximately 18.6 in Europe, 18.0 in Asia, 10.2 in Latin America and 7.4 in Africa (South Africa). However, the prevalence of AS is extremely high in certain countries, such as Turkey (11.9–49.0), China (37.1 specifically in the Shenzhen area), Italy (37.0), Taiwan (33.7) and the USA (31.9) [2]. AS has a significant correlation with human leukocyte antigen B27 (HLA-B27). The prevalence of HLA-B27 in the AS population is higher than 90% [3]. A recent study suggested that upregulation of the tissue-nonspecific alkaline phosphatase (TNAP)-related pathway caused by misfolding of HLA-B27 may contribute to the abnormal osteogenesis of AS in both a cell model and animal model. Additionally, the therapeutic potential of agents inhibiting TNAP was shown, though some adverse effects have been reported [4, 5].

HLA-B27 is considered one of the most important genetic factors contributing to AS. However, only 1–2% of HLA-B27 carriers develop AS, so HLA-B27 is not always reliable as a diagnostic or prediction criterion [3]. Furthermore, populations with a higher AS prevalence do not have a significantly higher ratio of HLA-B27 carriers. The prevalence of HLA-B27 carriers is approximately 10% among Caucasians, 8% among Han Chinese and 6% among the general population in Taiwan [6]. Previous familial aggregation studies indicated that heritability affects a considerable proportion of individuals with AS susceptibility [7]. Furthermore, ethnicity-specific genetic factors might be associated with disease severity and the high prevalence in certain populations [8, 9].

Genome-wide association studies (GWASs) are used to investigate correlations between genetic variants and traits of interest, especially associations between SNPs and diseases [10]. In the past decade, several GWASs have investigated the risk SNPs associated with AS, and hundreds of risk SNPs have been identified [11–20]. One risk AS-associated SNP, rs17192932, is specific to the Turkish population, which has a low prevalence of HLA-B27; this suggests the existence of ethnicity-specific risk SNPs in certain populations [19]. To date, no GWAS has been performed to study AS in individuals of Taiwanese descent.

The Taiwan Precision Medicine Initiative (TPMI) has recruited volunteers to collect Taiwanese genetic data and develop precision-based medicine since 2018. We used data from Tri-Service General Hospital (TSGH), which has joined the TPMI, to perform a GWAS to investigate risk SNPs associated with AS in the Taiwanese population.

We aimed to explore the race-specific AS susceptibility SNPs in Taiwanese individuals and to investigate the association between HLA-B27 and the AS susceptibility SNPs in Taiwan.

## Methods

### Ethics

The protocol of this study was reviewed and approved by the Institutional Review Board of the Tri-Service General Hospital (NO.: B202005140).

### Study participants and genotyping

All the participants employed in this study were recruited from TSGH to join the TPMI project. TPMI is held by Academia Sinica with a partnership of 15 top medical centers in Taiwan and aims to establish a database consisting of comprehensive clinical data and genetic profiles of one million participants. Participants were recruited from medical centers and genotyped by Academia Sinica. Briefly, approximately 5 mL of peripheral blood per participant was collected into EDTA vacutainers; genomic DNA was extracted from mononuclear cells and genotyped by TPMI SNP array following TPMI’s regular protocol. The TPMI SNP array is modified from Axiom Genome-Wide TWB (Taiwan Biobank) 2.0 Array Plate, can test approximately 130 thousand known risk variants, 580 thousand mapping SNPs and 20 thousand copy number variant markers based on Taiwanese reference genome data and Taiwan Biobank whole genome sequencing data. This TPMI project only recruited Taiwanese people of which the Southern Han population accounted for 95% and the Indigenous populations accounted for 2.3% [21].

Participants were allotted into two subcohorts based mainly on two batches of genotyping work. Assignment to the case or control group was based on the SOAP (The Subjective, Objective, Assessment and Plan) notes by clinicians. Participants diagnosed with AS were assigned to the case group, and the rest of the participants excluding those recruited from the Rheumatology, Immunology and Allergy Division were assigned to the control group to avoid sample contamination by those who were potentially but not yet diagnosed with AS. A total of 1442 participants, including 206 AS cases and 1236 normal participants, were enrolled in this study. There were 120 AS cases and 720 controls in the first batch and 86 AS cases and 516 controls in the second batch.

### Association analysis

The quality control and association analysis was conducted by PLINK 1.9 [22]. Quality control was carried out following the pipeline of automated quality control [23]. Individuals with a missing rate of genotype data above 0.05, indicating poor genotype quality, and were excluded. The study population contained individuals with high relatedness, which may result in bias. Individuals were excluded if they had a heterozygosity rate higher than three standard deviations of the means or an identity-by-descent (IBD) higher than 0.1875, which is the median value between second- and third-degree relatives. Then, the sex and age factor were adjusted by a regression analysis. SNPs with call rates lower than 0.95, Hardy–Weinberg equilibrium P values lower than 1 × 10^–4^, or minor allele frequency (MAF) lower than 0.05 were excluded. SNPs with P values in association analysis lower than 5 × 10^–8^ were considered significant. The Manhattan plot and Quantile–Quantile plot were generated by the R package qqman. The susceptibility of SNP-AS was adjusted to sex and age variables by logistic regression conducted with PLINK 1.9 and is presented as an odds ratio with a 95% confidence interval. The command code can be found in GitHub project (https://github.com/LinWZ-tw/PLINK_AS_GWAS.git).

### Functional genomics study of AS-associated SNPs

To further investigate the function or the pathogenic mechanism of risk SNPs, their locations and mapped genes were sorted from SNPnexus (https://www.snp-nexus.org/v4/).

The linkage disequilibrium (LD) of risk SNPs was analyzed by Haploview 4.2, and the LD pattern was generated by a web-based tool, LDmatrix (https://ldlink.nci.nih.gov/?tab=ldmatrix). The most significantly associated SNPs (with the lowest p value) were selected from each haplotype block as tag SNPs. The expression of genes mapped by tag SNPs was obtained from the Genotype-Tissue Expression (GTEx) portal, which was based on samples from donors in the United States [24]. The expression levels in skeletal muscle, fibroblasts, and whole blood were measured.

### Polygenic risk score prediction

We computed a set of polygenic risk scores (PRSs) derived from the genotype data of combined cohorts following the guidelines reported previously [25]. In brief, we executed six mainstream PRS methods, including Clumping and Thresholding [26], Lassosum [27], LDPred2 [28], GenEpi [29], PRS-cs [30], and PRSice [31], following their protocols and default settings. To perform a fair evaluation in the PRS prediction, we performed the train-test split by first randomly selecting ten percent of the data as an independent testing dataset for the prediction performance evaluation. Next, we applied quality control to the training dataset from ninety percent of the data. We then performed GWAS analysis by PLINK and built the PRS models using different methods on the training dataset. Of note, all methods except GenEpi used the same GWAS summary statistics as the starting point but selected different SNPs for inclusion in the prediction models according to their algorithms by tuning parameters. Because GenEpi applies a machine learning-based approach to include pairwise epistasis of genes, GenEpi makes choices differently for which SNPs to include in comparison with the other five methods. Finally, the area under the receiver operator characteristic (ROC) curve and the odds ratio of each different PRS decile relative to the rest of the data was calculated for the testing dataset to compare the performances of the six PRS models.

To examine whether model predictive ability is linked to elevated disease risk among individuals with high PRSs, we constructed a strata plot according to the PRS decile used in several previous studies [32–34]. In essence, the testing cohort was divided into 10 strata of increasing PRS estimated by each method. Then, we compared the prevalence percentage of each stratum to the whole testing cohort to obtain odds ratios of the risk for developing AS.

### Statistical analysis

SPSS 22.0 (IBM Crop.) and Excel 365 were used for all the data preparation, processing and analysis. Categorical variables are presented as counting numbers or percentages. Continuous variables are presented as the mean value with the standard error. Proportions with a two-tailed *P* value of less than 0.05 were considered statistically significant. The differences in variables by type were estimated by the chi-square test or t test. The proportions of SNPs interacting with HLA-B27 were estimated by the chi-square test and confirmed by logistic regression.

## Results

### Sample selection and characterization

**Fig. 1:**
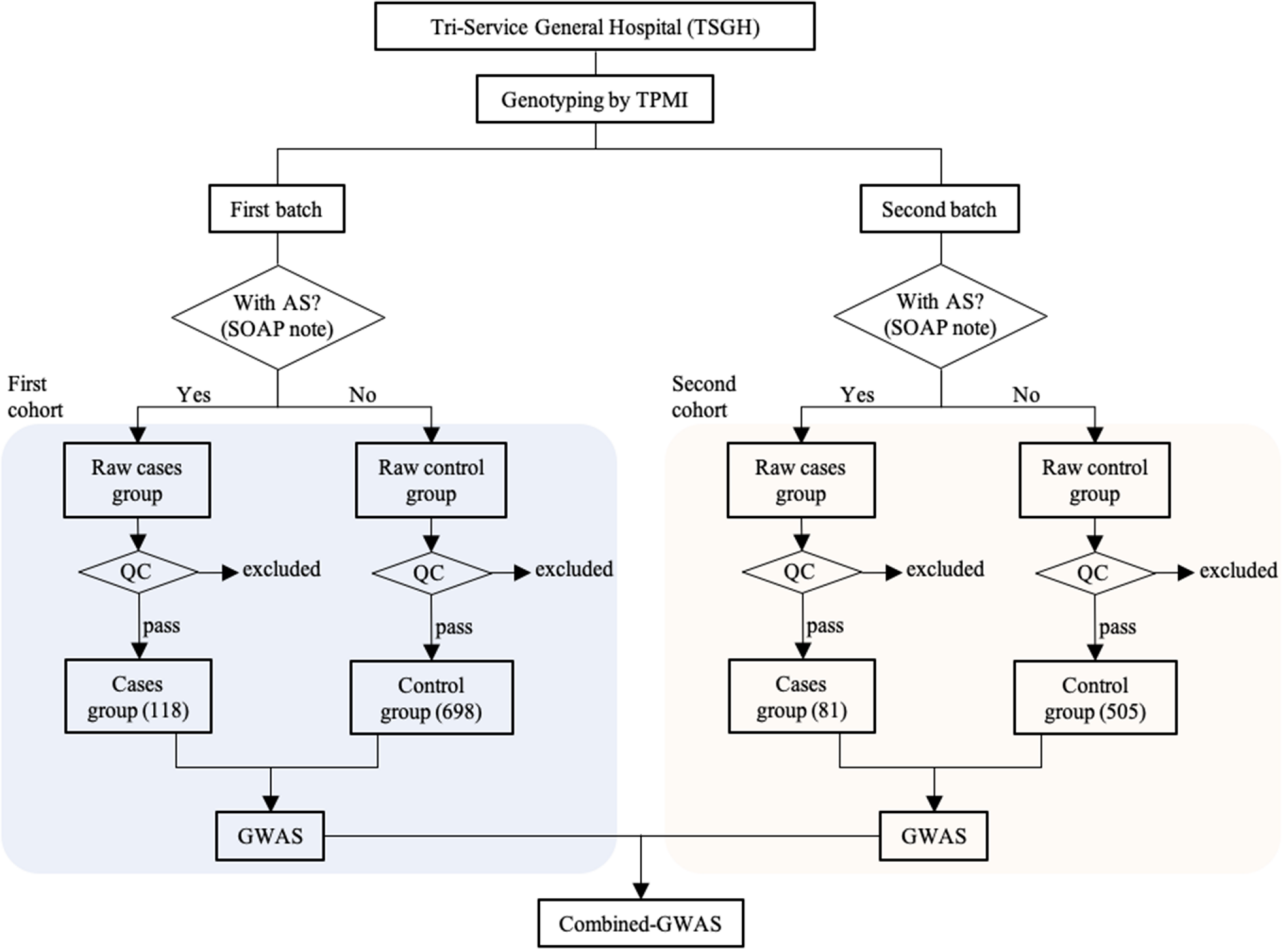
Flowchart of the study

After quality control, 1402 participants were enrolled, including 118 AS patients and 698 non-AS participants in the first batch and 81 AS and 505 normal participants in the second batch. Twenty-four participants were excluded in the first batch (3 had a high missing rate, 8 had a high heterozygosity rate, and 13 had a high IBD), and 16 participants were excluded in the second batch (7 had a high heterozygosity rate, and 9 had a high IBD) (Fig. 1). There were 88 (74.6%) male and 30 (25.4%) female AS patients in the first batch and 61 (75.3%) male and 20 (24.7%) female AS patients in the second batch. The mean ages were 39.63 (± 11.66) and 39.32 (± 13.95), respectively. (Table 1).

**Table 1:** Characterization of participants

Baseline Variable	GWAS study		P value	Replication Study		P value	P value	
	Case	Control		Case	Control		Case	Control
	n = 118 (%)	n = 698 (%)		n = 81 (%)	n = 505 (%)		0.936	0.113
Sex			9.23E-09			4.24E-08		
Male	88 (74.6)	321 (46.0)		61 (75.3)	209 (41.4)			
Female	30 (25.4)	377 (54.0)		20 (24.7)	296 (58.6)			
Age (y, mean ± sd)	39.63 ± 11.66	49.94 ± 16.11	1.04E-14	39.32 ± 13.95	44.72 ± 16.63	0.001	0.762	6.54E-08
Age (y) Range			1.26E-10			0.018	0.102	6.12E-10
< 20	0 (0.0)	12 (1.7)		0 (0.0)	9 (1.8)			
20–29	20 (16.9)	68 (9.7)		25 (30.9)	102 (20.2)			
30–39	42 (35.6)	112 (16.0)		24 (29.6)	116 (23)			
40–49	34 (28.8)	139 (19.9)		16 (19.8)	77 (15.2)			
50–59	12 (10.2)	173 (24.8)		8 (9.9)	77 (15.2)			
60–69	10 (8.5)	123 (17.6)		6 (7.4)	96 (19.0)			
≧70	0 (0.0)	71 (10.2)		2 (2.5)	28 (5.5)			
HLA-B27 ( +)^a^	76 (77.6)	–	–	56 (73.7)	–	–		

^a^Only the patients in the case group were tested for HLA-B27; the patients in the control group lacked data

### AS-associated SNPs in the taiwanese population

**Fig. 2:**
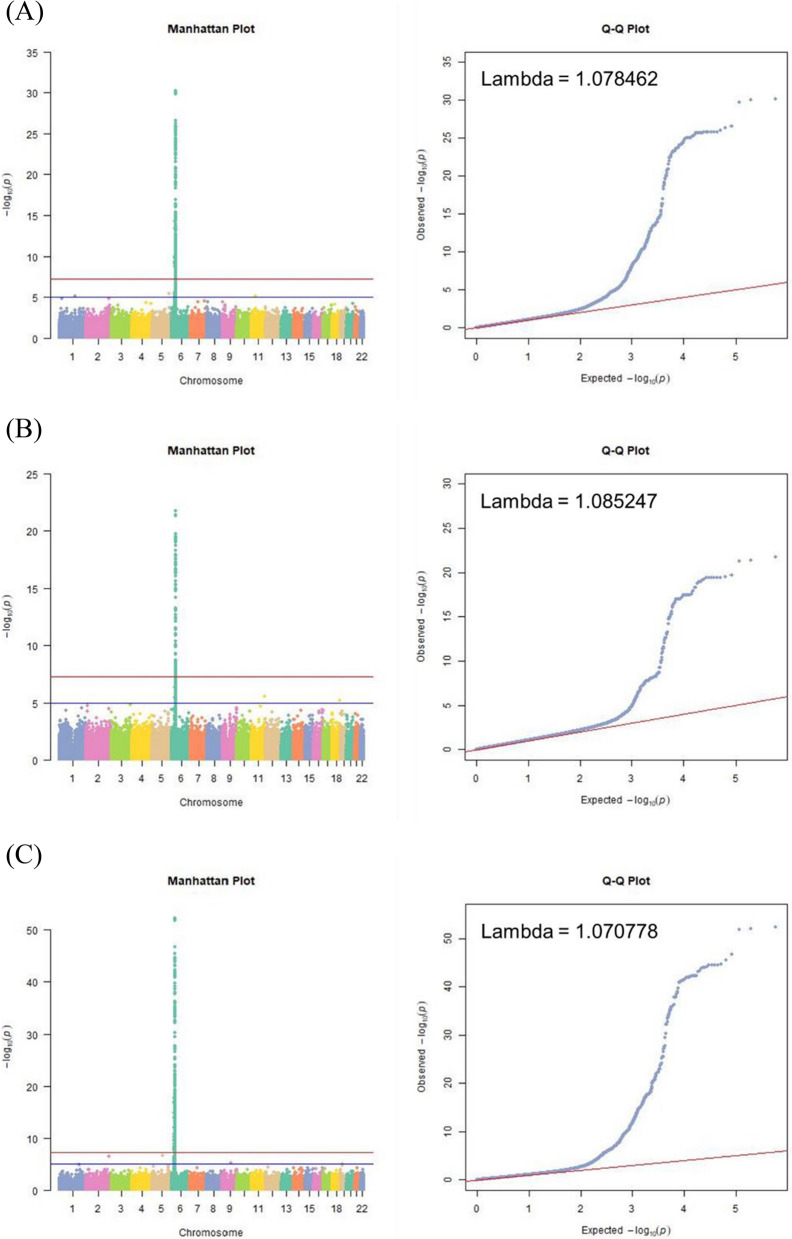

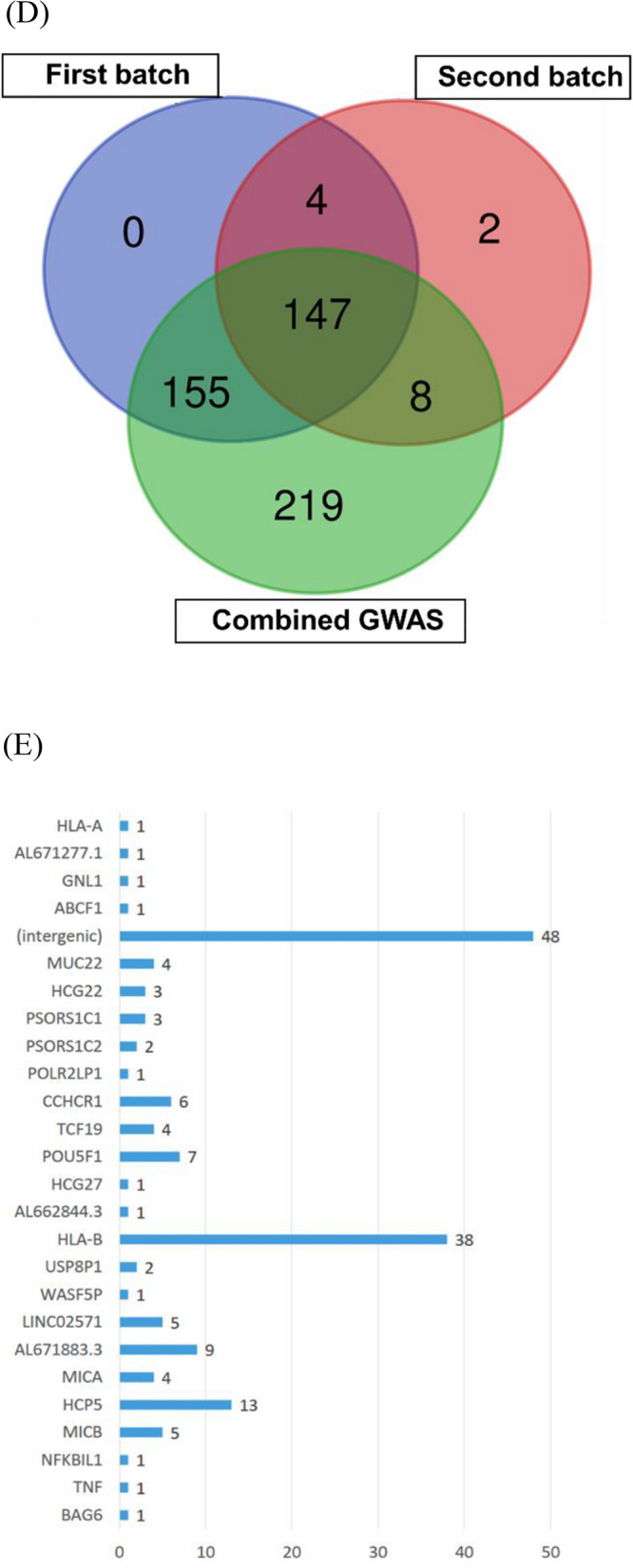

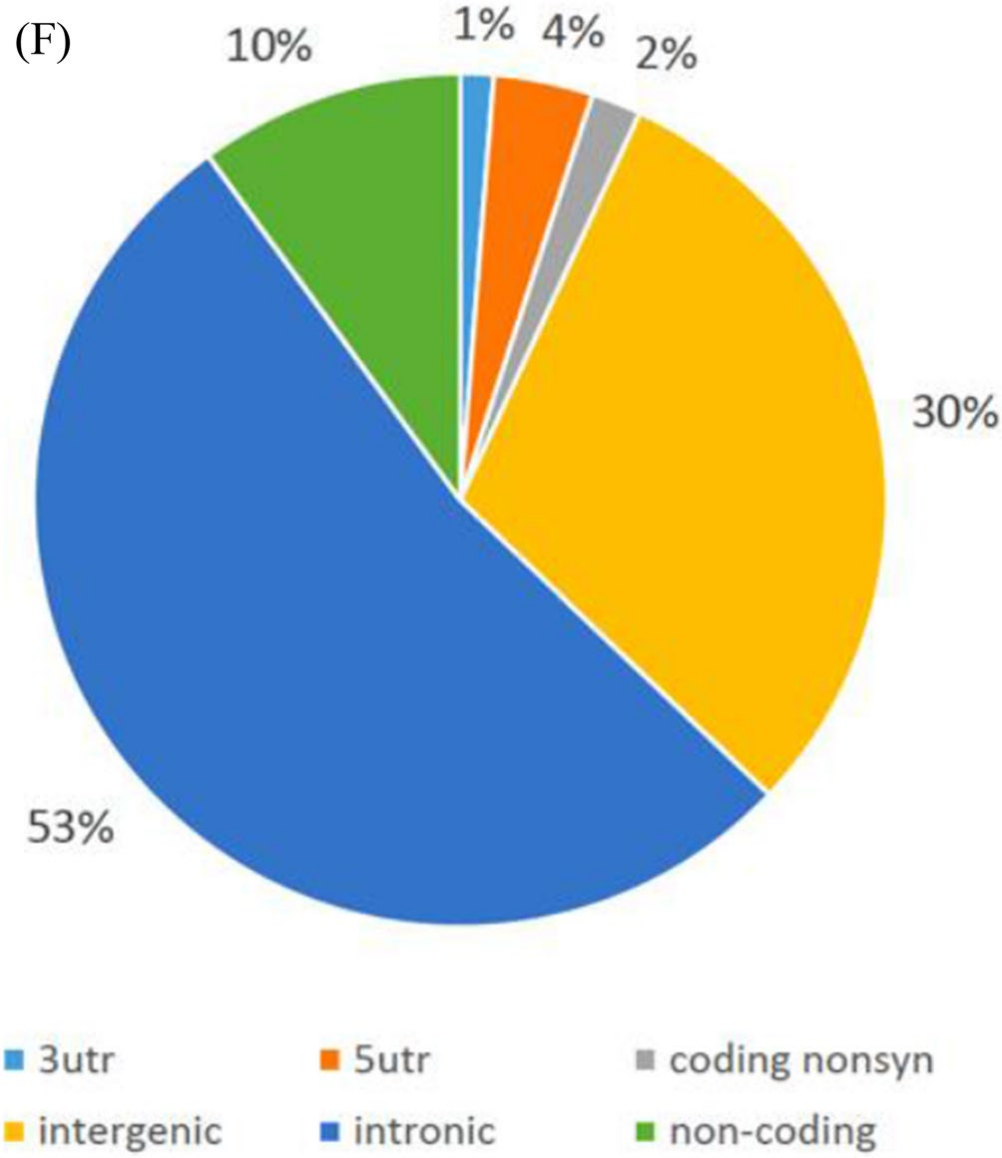
AS-associated SNPs in the Taiwanese population. **A** The GWAS results presented as a Manhattan plot and QQ plot for the first cohort, (**B**) the second cohort and (**C**) the combination cohort. **D** The associated SNPs were picked up from a Venn diagram of the three association studies. **E** The locations of 147 SNPs raised in three association studies and their mapped genes. **F** The ratios of the SNP corresponding genes. Coding nonsyn: nonsynonymous coding region

There were 147 AS-associated SNPs raised from two batches of association studies and the combined batch (Additional file 1: Table S1). The results of the first, second and third batches all showed that the significant AS-associated SNPs were clustered on chromosome 6 (Fig. 2A–C). The lambda values ranged from 1.07 to 1.09, which may indicate a low risk of or insufficiently corrected population stratification. (Fig. 2A–C).

### AS-associated genetic loci and corresponding genes

In total, 306 loci were found to be significant in the association analysis of the first batch (P < 10^–8^). A total of 161 loci were found to be significant in the second batch (P < 10^–8^). A total of 529 loci were found to be significant in the combined batch (P < 10^–8^). A Venn diagram was used to find the intersection. A total of 147 loci were found to be the most obviously related loci (Fig. 2D).

The 147 loci were annotated to their corresponding genes by the Ensemble and SNP nexus (Fig. 2E). Some SNPs may be annotated to more than one gene; therefore, the sum of SNP numbers by each corresponding gene exceeded 147.

The loci at introns accounted for 53%, the loci at intergenic regions accounted for 30%, the loci in noncoding regions accounted for 10%, the loci at the 5’UTR accounted for 4%, the loci at the nonsynonymous coding region accounted for 2%, and the loci at the 3’UTR account for 1%. (Fig. 2F).

### Functional genomics of AS-associated SNPs

**Fig. 3:**
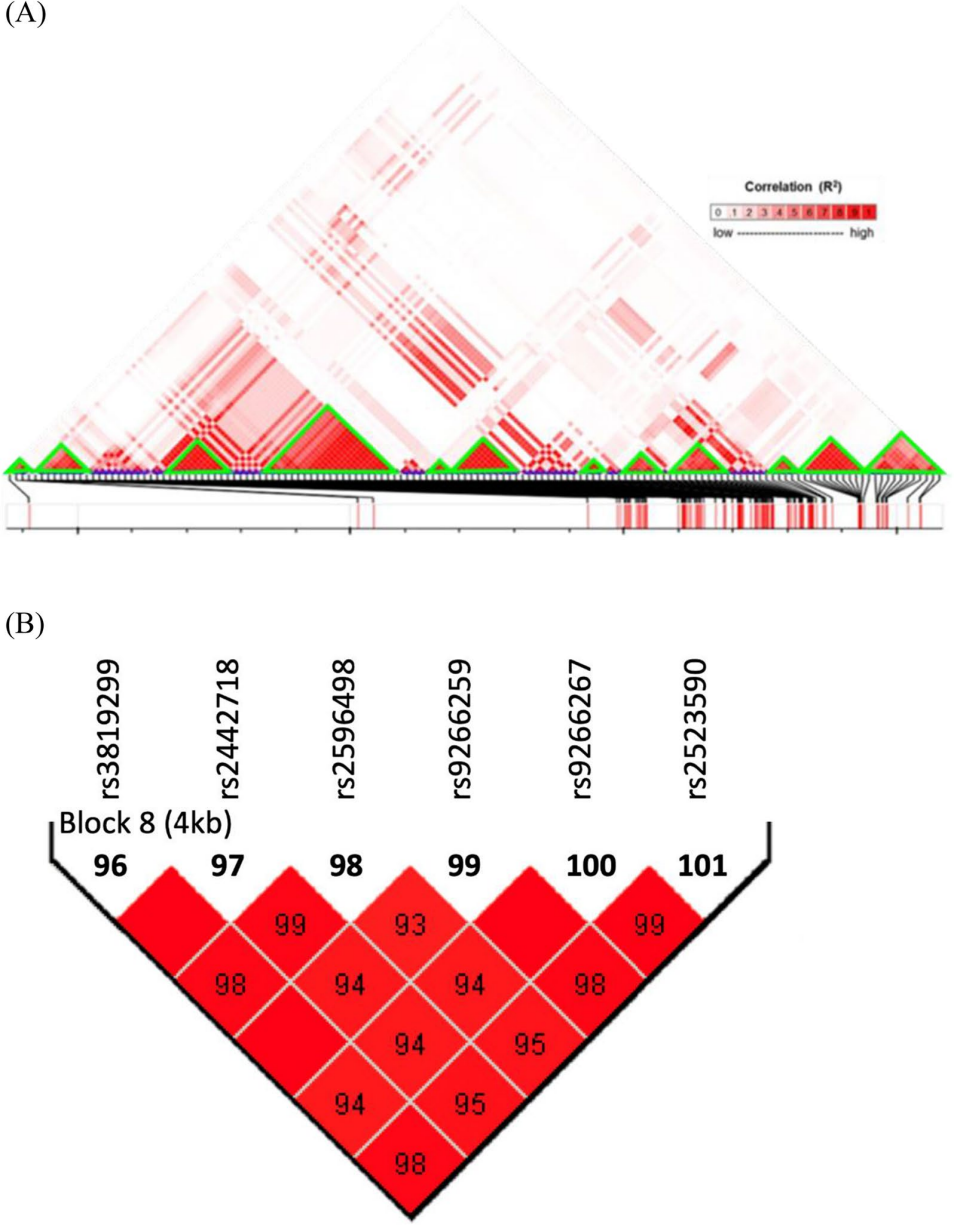
Functional genomics of AS-associated SNPs. **A** A total of 147 AS-associated SNPs were assigned to 12 haplotype blocks. **B** Haplotype Block 8. rs9266267 is the tag SNP in haplotype block 8

The LD analysis suggested that the 147 AS-associated SNPs could be assigned into 12 haplotype blocks. The Haploview program was used to exclude SNPs covering multiple bases in at least one allele. (Fig. 3A).

The locus with the smallest p value in every haplotype block was used as the tag SNP. These 12 tag SNPs are the most representative loci of the 12 haplotype blocks. (Table 2).

**Table 2:** The tag SNPs of each haplotype block

Block	SNP	Gene	A1/A2^a^	MAF	MAF case	MAF control	MAF CHS	OR^b^	95% CI^b^	P value^b^
1	rs142577772	GNL1	T/C	0.085	0.297	0.049	0.033	11.36	(7.18–15.12)	1.45E-34
		(3’ UTR)								
2	rs7756294	–	A/G	0.131	0.381	0.089	0.067	8.31	(5.95–11.76)	7.48E-33
		(intergenic)								
3	rs2073716	CCHCR1	G/C	0.206	0.432	0.168	0.186	3.92	(2.79–4.80)	7.56E-21
		(intron)								
4	rs76977405	–	G/T	0.110	0.372	0.066	0.043	15.27	(9.27–19.45)	1.67E-42
		(intergenic)								
5	rs7766452	HLA-B	A/G	0.088	0.372	0.041	0.014	34.17	(17.73–41.22)	1.72E-51
		(intron)								
6	rs9368671	HLA-B	T/C	0.300	0.504	0.266	0.219	3.40	(2.54–4.33)	9.82E-19
		(intron)								
7	rs28862571	HLA-B	T/C	0.151	0.415	0.107	0.086	10.14	(6.43–12.75)	2.35E-36
		(intron)								
8	rs9266267	–	C/A	0.123	0.392	0.078	0.057	20.68	(13.20–29.62)	8.23E-46
		(intergenic)								
9	rs6936035	AL671883.3	G/A	0.286	0.556	0.241	0.195	4.46	(3.81–6.83)	7.33E-25
		(intron)								
10	rs2251396	MICA	A/G	0.314	0.517	0.280	0.286	3.33	(2.37–3.94)	6.43E-19
		(intron)								
11	rs3094228	HCP5	C/T	0.229	0.458	0.190	0.138	4.36	(3.19–5.65)	1.19E-22
		(intron)								
12	rs9688839	HCP5	G/A	0.169	0.364	0.136	0.086	4.18	(2.74–4.78)	1.07E-21
		(intron)								

^a^A1 = minor allele, A2 = major allele

^b^The ORs, 95% CIs and P values presented in the table are calculated based on data from the combined study

Three tag SNPs located in intergenic regions were not further studied, and the other nine tag SNPs were mapped to GNL1, CCHCR1, HLA-B, AL671883.3, MICA, and HCP5, among others. (Table 2) Nine of the 12 tag SNPs in the haplotype blocks corresponded to genes: (Table 2) GNL1: rs142577772 (OR = 11.36, p value = 1.45E-34); CCHCR1: rs2073716 (OR = 3.94, p value = 3.99E-21); HLA-B: rs7766452 (OR = 34.17, p value = 1.72E-51); rs9368671 (OR = 3.40, p value = 9.82E-19); rs28862571 (OR = 10.14, p value = 2.35E-36); AL671883.3: rs6936035 (OR = 4.46, p value = 7.33E-25); MICA: rs2251396 (OR = 3.33, p value = 6.43E-19); HCP5: rs3094228 (OR = 4.36, p value = 1.19E-22); and rs9688839 (OR = 4.18, p value = 1.07E-21). The other 3 tag SNPs were located at the intergenic region, so there was no corresponding gene.

The tag SNPs were correlated with the expression of HLA-C, HLA-B, HLA-S, MICA, MICB, CCHCR1, HCG20, HCG27, MIR6891, NCR3, PSORS1C3, C4A, C4B, CYP21A1P, CYP21A2, DDAH2, GLOT1, LINC00243, LY6G5B, NOTCH4, PPP1R18, RNF5, XXbac BPG248L24.12 and XXbac BPG181B23.7 in skeletal muscle, fibroblasts or whole blood (Additional file 2: Table S2). In Additional file 2: Table S2, A1 is the minor allele and A2 is the major allele. Normalized effect size (NES) is the value of the alternative allele/reference allele. Positive values indicate that the alternative allele is correlated with increased expression level, and vice versa.

### Association between HLA-B27 and genotype with minor allele

The results of the association between HLA-B27 and the genotype with the minor allele are presented in Table 3. rs7766452 had the highest odds ratio (OR), and the 95% confidence interval (CI) of OR was 56.69–1317.97. This means that AS patients with minor allele genotypes (AG, AA) are 273.33 times more likely to be HLA-B27 positive than AS patients without minor allele genotypes (CC).

**Table 3:** Association between HLA-B27 and tag SNPs

SNP	OR	95% CI	P value
rs142577772 (ref:CC)	26.91	(7.87–92.00)	1.53E-07
rs7756294 (ref:GG)	38.00	(14.02–102.99)	8.63E-13
rs2073716 (ref:CC)	17.85	(7.64–41.70)	2.82E-11
rs76977405 (ref:TT)	48.71	(16.79–141.30)	8.63E-13
rs7766452 (ref:GG)	273.33	(56.69–1317.97)	2.74E-12
rs9368671 (ref:CC)	27.56	(9.92–76.58)	2.03E-10
rs28862571 (ref:CC)	38.75	(14.55–103.19)	2.51E-13
rs9266267 (ref:AA)	NA	NA	NA
rs6936035 (ref:AA)	94.12	(20.45–433.16)	5.39E-09
rs2251396 (ref:GG)	19.09	(6.89–52.87)	1.39E-08
rs3094228 (ref:TT)	32.00	(12.68–80.73)	2.13E-13
rs9688839 (ref:AA)	15.79	(6.57–37.90)	6.68E-10

With rs9266267 being the exception, 11 tag SNPs were statistically significantly associated with HLA-B27 positivity (p value < 0.05). It might be inferred that “whether HLA-B27 is positive or not” is directly related to “whether the genotype has the minor allele genotype”.

### Polygenic risk score differentiated patients with AS from controls

**Fig. 4:**
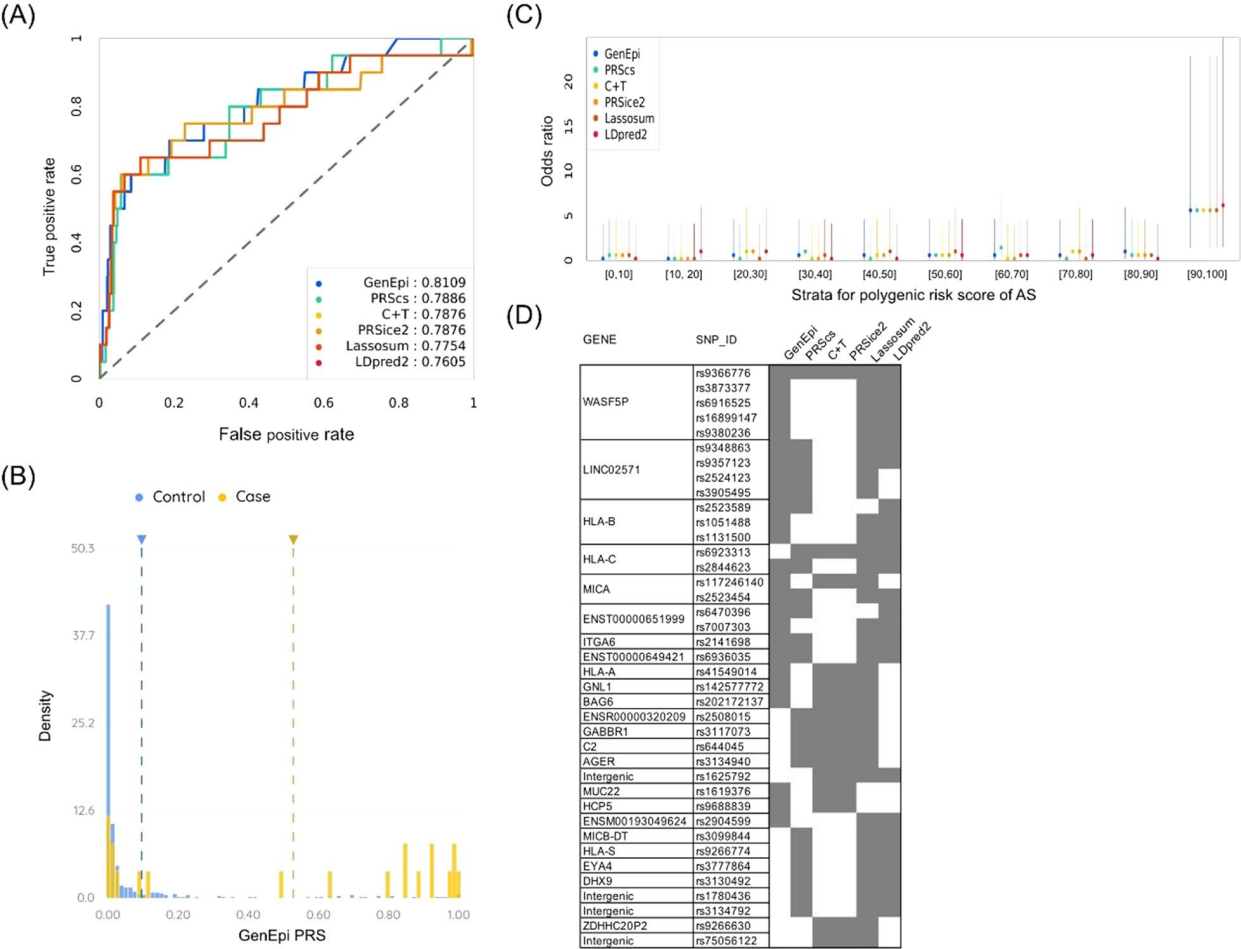
Prediction of the polygenic risk score of AS. **A** Receiver operating characteristic (ROC) curve of the polygenic risk score (PRS) for the prediction of AS. The areas under the ROC curves (AUROCs) for the six PRS methods are reported in the figure legend box. **B** The distribution of PRSs of the AS cases and controls predicted by the GenEpi method (the blue dashed line represents the median PRS of the control group, and the yellow dashed line represents the median PRS of the case group). **C** Strata plot of the odds ratio for developing AS generated by different PRS methods. The X-axis shows the range of the decile quantiles, and the Y-axis shows the odds ratio when comparing the PRS of corresponding quantiles (e.g., [90, 100] corresponds to those individuals with PRSs between the 90th and 100th percentile of the cohort population) with the rest of the PRS quantiles of individuals. The bars show the 95% confidence intervals of the odds ratios. **D** The annotated genes with the consensus SNPs selected by at least three PRS methods in the trained AS-PRS models. The gray blocks indicate that the SNPs were used in the prediction model of the given methods

We developed several PRS models on the genotyping data of AS and control cohorts using six different methods (Fig. 4). According to the area under ROC curves on the independent testing dataset, GenEpi achieved the highest performance with an AUROC of 0.8109, and LDpred2 yielded the lowest performance with an AUROC of 0.7605 (Fig. 4A). Interestingly, among 227 SNPs used by GenEpi to estimate the PRS of developing AS, there were 110 SNP pairs and 7 single SNPs (Additional file 3: Table S3). The distribution of scaled GenEpi PRS remarkably differed between the AS case and control groups (Fig. 4B). A similar trend of AS-PRS distributions between the case and control groups was observed for the other five methods (Additional file 5: Figure S1). In addition, the strata plot indicates that the top decile showed a striking increase in the prevalence percentage in the last stratum (Additional file 5: Figure S2). In addition, the highest ORs of developing AS in the last stratum compared with the whole testing cohort were observed in all six methods (Fig. 4C). That is, individuals in the top PRS decile had over a fivefold increased risk of being diagnosed with AS compared to those in the lower PRS deciles (Additional file 4: Table S4).

Finally, we selected a set of SNPs used by at least three out of six methods as the important features in the trained AS-PRS models. Despite the diverse procedures in tuning PRS models for different algorithms, twenty-three human genes with consensus SNPs contributing to different PRS models are shown (Fig. 4D).

## Discussion

This is the first GWAS of a Taiwanese AS population analyzed with the TPMI database. The association study followed a typical protocol for GWAS. We conducted two association studies based on two batches of genotyping data and one based on a dataset merging the two batches of genotyping data. Only the SNPs (n = 147) raised in three associated studies were considered AS-associated SNPs. The SNPs located on the sixth chromosome had higher susceptibility in the AS group. There was no overlap between our results and the susceptibility SNPs found in people of other races. These 147 AS-associated SNPs were assigned to 12 haplotype blocks. The SNP with the lowest p value among every haplotype block was considered the tag SNP. Nine tag SNPs corresponded to genes, and 11 tag SNPs had statistically significant associations between HLA-B27 and genotypes with minor alleles.

The precise pathogenesis of AS is still unknown. However, this autoimmune disease is related to multifactor interactions, such as genetic background, immune response, environmental factors, and microbial infection [3]. Since 1961, AS has been known to involve a non-sex-linked dominant hereditary mechanism [35]. Genetic effects have been identified as causative factors, accounting for more than 90% of the population variation [36]. Previous studies indicated that the major histocompatibility complex (MHC) on chromosome arm 6p and HLA-B27, one of the MHC-1 molecules, is strongly linked to and associated with AS [37, 38]. This is compatible with our results. Although approximately 95% of Caucasian patients with AS are HLA-B27 positive, only 8% of HLA-B27-positive individuals in the population develop the disease [39–41]. This means that HLA-B27 is essential for family inheritance but that there are still other genetic risk factors. HLA-B27 has a high degree of genetic polymorphism, and more than 100 known subtypes have been identified. The distinct subtypes are related to the prevalence of AS in the different regions of the world. The most significant subtypes associated with AS are HLA-B*27:05 (Caucasians), HLA-B*27:04 (Chinese), and HLA-B*27:02 (Mediterranean populations) [42, 43]. Laval’s whole-genome screening study indicated that genes localized to chromosomes 1p, 2q, 6p, 9q, 10q, 16q, and 19q were associated with AS [44].

GWASs have been used to map the patterns of inheritance for the SNP, the most common form of genomic variation [45, 46]. A GWAS in 2010 surveying AS in a large population of European descent revealed that multiple gene variants, including ARTS1, IL23R, ANTXR2 and IL1R2, confer AS risk [47]. In the past decade, the following GWASs have identified 113 SNPs affecting the risk of developing AS. Furthermore, an ongoing GWAS will likely identify more than 100 new risk loci [14, 17, 48]. However, GWASs of the Han Chinese AS population are few [12, 49], and no GWAS has been performed among the Han Taiwanese AS population. A previous study indicated that ethnic differences would lead to genetic heterogeneity in AS susceptibility. Some genes, including those in the 2p15, ERAP1, and NPEPPS–TBKBP1 regions, may still play a critical role in AS pathogenesis across diverse populations [50].

Our results revealed that the AS-associated SNPs were clustered around HLA-B27. While many of them were located in intergenetic regions (30%), the others could be mapped to a group of genes. Among them, 38 SNPs were mapped to HLA-B, and some were mapped to HCP5 (13 SNPs), AL671883.3 (9 SNPs), POU5F1 (7 SNPs), CCHCR1 (6 SNPs), LINC02571 (5 SNPs), MICB (5 SNPs), MUC22 (4 SNPs), TCF19 (4 SNPs), and MICA (4 SNPs), among others. (Fig. 3).

Data collected from GTEx Portal reveal that SNPs are associated with the expression level of their mapped genes. The tag SNP, rs142577772, of Haplotype block 1 is located in the 3 prime UTR of the GNL1 gene. The mutation position of rs142577772 is the CCCTC-binding factor binding site. This multifunctional transcription regulator might affect the expression of multiple epigenes [51]. The GNL1 gene and HLA-E gene present a high degree of linkage disequilibrium. There is a strong association between the HLA-E gene and AS haplotype [52]. It could be inferred that the GNL1 gene might be associated with AS. The tag SNP, rs2073716, of haplotype block 3 is located in the intron of the CCHCR1 gene. The CCHR1 locus may be protective against AS [53]. rs7766452 of haplotype block 5, rs9368671 of haplotype block 6 and rs28862571 of haplotype block 7 are located in introns of the HLB-B gene. The HLA-B gene is listed as an AS-related gene in the GWAS Catalog database. A GWAS of Turks and Iranians indicated that rs17192932, HLA-B*2705, HLA-B*2702 and HLA-B2707 are variants of HLA-B related to AS [19]. rs6936035 of haplotype block 9 is located in the intron of the AL671883.3 gene. The AL671883.3 gene was shown to be an AS-related gene in the GWAS Catalog database and previous studies [11, 14]. rs2251396 of haplotype block 10 is located in the intron of the MICA gene. The MICA gene is listed as an AS-related gene in the GWAS Catalog database and a previous study [13]. rs3094228 of haplotype block 11 and rs9688839 of haplotype block 12 are located in the introns of the HCP5 gene. Coit’s study indicated that the genetic variant present in the CpG methylation site in HCP5 determines its methylation status and is linked to HLA-B*27 status in AS patients [54].

HLA-B27 is a necessary factor for the development of AS. However, only 77.6% of subjects in our first batch and 73.7% of subjects in our second batch were HLA-B27 positive. The minor allele frequency in the case group was significantly higher than that in the South Han Chinese population (Additional file 2: Table S5). The genotype distribution of the three SNPs (rs2524069, rs2524067 and rs7766452) with the smallest p value in haplotype block 5 showed that the proportion of HLA-B27-positive people with minor alleles was higher than that of HLA-B27-negative people. Most people with HLA-B27 positivity carry only one minor allele. Most people who are HLA-B27 negative carry the major allele. This might infer that the proportion with minor alleles in the SNP is relatively high in the Taiwanese population. This would cause the prevalence of AS to be higher than that in other regions of the world, but the proportion of HLA-B27 is not higher.

In addition, we used six PRS methods to estimate the risks of developing AS in Taiwanese populations. Overall, six PRS models yielded good performance with an AUROC of approximately 0.76 (Fig. 4A), and the top 10% of PRSs showed at least a fivefold increase in developing AS compared to the remaining lower risk groups (Fig. 4C). It is noteworthy that we employed the train-test split method to evaluate the PRS models using the independent testing cohort to avoid the overfitting problem. Among the six AS-PRS models, the GenEpi model achieved the highest performance in terms of its AUROC value (Fig. 4A). In contrast to the other five methods, GenEpi applies a machine learning approach to identify the epistasis effect of joint genetic effects associated with AS. Indeed, GenEpi identified 110 significant SNP-SNP interactions across entire genomic loci harboring many different genes (Additional file 2: Table S2). The most significant interaction effect on AS was found between two SNPs, rs2844532 (near HLA-S gene) and rs2904599 (near MICA gene), with a *p* value of 1.628 × 10^–125^. Both the HLA-S and MICA genes have been implicated to have SNPs associated with AS in a previous study, whereas the joint effect of two SNPs within these two genes has not yet been suggested [13]. On the other hand, we identified several genomic loci, including 23 genes, as having consensus SNPs that contributed considerably to at least three PRS models (Fig. 3D). Among 23 genes, many have been suggested to be associated with AS and other related autoimmune diseases, such as psoriasis and vitiligo, in GWASs. For example, WASF5P and LINC02571 have both been reported to be significantly associated with vitiligo in the Chinese Han population by GWAS [55]. Additionally, HLA-B, HLA-C, and MICA are indeed AS-related genes, as discussed in the aforementioned paragraphs. Since six different PRS methods apply different algorithms to evaluate the importance of SNPs to estimate the risk of developing AS, the genes with consensus SNPs could be considered critical genetic information related to AS. Therefore, the genomic loci in Fig. 4D might potentially be used as amplicon-based genes for the prediction of AS.

Several limitations exist. First, the data are from the division of rheumatology of a single medical center of the TPMI. As a result, there might be some AS patients going to other hospitals due to non-AS medical problems and joining the TPMI. These AS patients might be classified as a control group, which would cause bias. Second, we obtained the data from medical records. These data lack detailed basic demographic variables, personal health behaviors and living environment exposure. In addition, this study lacks HLA-B27 genotyping data in a normal population because only patients with symptoms of AS had HLA-B27 detected. Therefore, we could not analyze the association between HLA-B27 and SNPs in the general population. While SNPs are likely to have an effect on their mapped genes, SNPs located in intergenic regions were not assigned to any genes to avoid error of prediction. Third, the AS susceptibility SNPs found in this study almost did not overlap with the related SNPs in the GWAS Catalog database. For some SNPs, there is no relevant information or gene expression data for the corresponding gene in the database of the GTEx portal. However, all data in the GTEx portal are from donors in the United States. This discrepancy might be due to ethnic differences. In addition, few GWASs have been performed among the Han Chinese population [12, 49]. Currently, there is no GWAS of AS populations in Taiwan. We have few previous data to compare.

## Conclusion

We found 147 SNPs in the Taiwanese population that were statistically significantly associated with AS on the sixth pair of chromosomes, and the SNPs could be divided into 12 haplotype blocks (Table S1). There were 9 SNPs related to susceptibility to AS. These SNPs did not overlap with previously published sites on the GWAS Catalog. The prevalence of tag SNPs in the Taiwanese population is higher than that in other Asian populations with “1000 genomes” as reference (Table S5), which may explain why the AS prevalence is higher in the Taiwanese population than in other Asian countries. Further study will be needed in which researchers employ the risk SNPs revealed by this study as diagnosis biomarkers for populations outside of Taiwan. Whether those genes mapped by AS-associated SNPs are involved in AS and what the pathogenic mechanism of the mapped genes is remain to be further studied.

### Data resources and software

Information on SNP genotyping can be found in TPMI (https://tpmi.ibms.sinica.edu.tw/www/en/). The association studies were conducted based mainly on PLINK 1.9 (https://zzz.bwh.harvard.edu/plink/) and R 3.6.1 (https://cran.r-project.org/). The LD of SNPs was analyzed by Haploview 4.2 (https://www.broadinstitute.org/haploview/haploview) and displayed by a web tool, LD Link (https://ldlink.nci.nih.gov/).

## Supplementary Information

**Additional file 1: Table S1.** The 147 SNPs associated with AS

**Additional file 2: Table S2.** The correlation of SNPs with gene expression in certain tissues. **Table S5.** The prevalence of tag SNPs in Asian populations.

**Additional file 3: Table S3**. Information on selected SNPs in the six different PRS models.

**Additional file 4: Table S4.** Odds ratio for developing AS according to PRS deciles.

**Additional file 5: Figure S1.** Comparison of AS polygenic risk between AS cases and controls. **Figure S2.** Strata plot with ten strata of increasing PRS versus prevalence (%) of developing AS.

## Data Availability

The datasets used and/or analyzed during the current study are available from the corresponding author on reasonable request.

## Abbreviations

AS: Ankylosing spondylitis
GWAS: Genome-wide association study
SNP: Single-nucleotide polymorphism
TPMI: Taiwan Precision Medicine Initiative
MAF: Minor allele frequency
IBD: Identity by descent
LD: Linkage disequilibrium
GTEx: Genotype-tissue expression
PRS: Polygenic risk scores
