# Supplementary material for: Genome-wide association study reveals ethnicity-specific SNPs associated with ankylosing spondylitis in the Taiwanese population
Source: J Transl Med. 2022 Dec 12;20:589. doi: 10.1186/s12967-022-03701-3 (PMC9746141; doi:10.1186/s12967-022-03701-3)
Supplement: Supplementary file 2 — Additional file 2: Table S2. The correlation of SNPs with gene expression in certain tissues. Table S5. The prevalence of tag SNPs in Asian populations. [file 12967_2022_3701_MOESM2_ESM.pdf]

Supplementary Tables. The correlations of tag SNPs with mapped gene expression
Table S1. The 147 SNPs associated with AS.

Additional file 1

Table S2. The correlation of SNPs with gene expression in certain tissues.

SNP	*A1/A2**	*Gene Symbol*	*Tissue*	*NES**	*P value*
rs7766452	A/G	HLA-C	muscle–skeletal	0.61	4.90E-09
		HLA-C	whole blood	0.26	2.70E-04
		HLA-C	cell-cultured fibroblasts	1	7.70E-14
rs9368671	T/C	CCHCR1	cell-cultured fibroblasts	-0.18	1.20E-06
rs2073716	G/C	CCHCR1	muscle–skeletal	-0.37	9.40E-06
		CCHCR1	whole blood	-0.3	7.60E-07
		CCHCR1	cell-cultured fibroblasts	-0.17	6.00E-05
rs6936035	G/A	HLA-B	cell-cultured fibroblasts	-0.2	3.90E-04
		MICA	cell-cultured fibroblasts	-0.78	3.20E-35
		XXbac-BPG181B23.7	muscle–skeletal	-0.79	2.10E-53
		XXbac-BPG181B23.7	whole blood	-0.72	2.70E-73
		XXbac-BPG181B23.7	cell-cultured fibroblasts	-0.87	1.70E-42
rs9688839	G/A	HCG27	whole blood	0.15	2.30E-05
		HCG27	cell-cultured fibroblasts	0.31	6.10E-06
		MIR6891	whole blood	0.54	1.50E-10
		NCR3	whole blood	-0.14	8.40E-06
		PSORS1C3	muscle–skeletal	0.39	1.50E-04
		PSORS1C3	whole blood	0.33	2.00E-06
rs2251396	A/G	MICA	muscle–skeletal	0.31	8.40E-12
		MICA	whole blood	0.25	6.40E-13
		MICA	cell-cultured fibroblasts	0.63	1.70E-25
		C4A	muscle–skeletal	-0.29	1.60E-09
		C4A	whole blood	-0.48	1.50E-16
		C4A	cell-cultured fibroblasts	-0.28	3.50E-11
		C4B	whole blood	0.47	2.60E-13

		C4B	cell-cultured fibroblasts	0.3	2.20E-13
		CCHCR1	whole blood	0.13	8.10E-05
		CYP21A1P	muscle–skeletal	-0.31	8.00E-08
		CYP21A1P	whole blood	-0.4	1.10E-12
		CYP21A1P	cell-cultured fibroblasts	-0.23	4.30E-05
		CYP21A2	whole blood	0.4	3.20E-10
		CYP21A2	cell-cultured fibroblasts	0.28	1.50E-05
		DDAH2	muscle–skeletal	-0.1	5.90E-06
rs3094228	A/G	FLOT1	whole blood	0.06	2.20E-05
		HCG20	cell-cultured fibroblasts	-0.32	3.20E-05
		HLA-C	muscle–skeletal	0.42	2.40E-16
		HLA-C	cell-cultured fibroblasts	0.46	1.50E-10
		HLA-S	whole blood	-0.27	3.70E-05
		LINC00243	cell-cultured fibroblasts	0.29	1.50E-04
		LY6G5B	cell-cultured fibroblasts	0.14	3.60E-04
		MICB	muscle–skeletal	0.34	1.90E-10
		MICB	cell-cultured fibroblasts	0.37	1.30E-12
		NOTCH4	muscle–skeletal	-0.2	1.50E-07
		PPP1R18	cell-cultured fibroblasts	-0.11	1.90E-05
		RNF5	muscle–skeletal	-0.12	1.80E-07
		XXbac-BPG248L24.12	whole blood	-0.24	1.50E-04
rs142577772	T/C	-	-	-	-
rs28862571	T/C	-	-	-	-

* A1 is the minor allele, and A2 is the major allele.

** Normalized effect size (NES) = Alternative allele/reference allele. Positive values indicate that the alternative allele is correlated with increased expression level, and vice versa.

Table S3. Information on selected SNPs in the six different PRS models.

Additional file 2

Table S4. Odds ratio for developing AS according to PRS deciles.

Additional file 3

Table S5. The prevalence of tag SNPs in Asian populations.

Block	SNP	MAF						
		TW	CHS	CDX	CHB	JPT	KHV	EAS
1	rs142577772	0.085	0.033	0.07	0.01	0.01	0.015	0.027
2	rs7756294	0.131	0.067	0.113	0.049	0.019	0.136	0.075
3	rs2073716	0.206	0.186	0.204	0.146	0.197	0.182	0.183
4	rs76977405	0.11	0.043	0.091	0.053	0.154	0.071	0.082
5	rs7766452	0.088	0.014	0.054	0.005	0.005	0.045	0.024
6	rs9368671	0.3	0.219	0.382	0.17	0.144	0.207	0.221
7	rs28862571	0.151	0.086	0.118	0.136	0.236	0.091	0.134
8	rs9266267	0.123	0.057	0.07	0.087	0.159	0.061	0.087
9	rs6936035	0.286	0.195	0.22	0.238	0.207	0.232	0.218
10	rs2251396	0.314	0.286	0.478	0.262	0.135	0.348	0.298
11	rs3094228	0.229	0.138	0.242	0.126	0.024	0.369	0.177
12	rs9688839	0.169	0.086	0.129	0.121	0.154	0.116	0.121

TW: Taiwan

CHS: Southern Han Chinese, China

CDX: Chinese Dei in Xishuangbanna, China

CHB: Han Chinese in Beijing, China

JPT : Japanese in Tokyo, Japan

KHV: Kinh in Ho Chi Minh City, Vietnam

EAS: East Asian
