# Supplementary material for: Genome-wide association study reveals ethnicity-specific SNPs associated with ankylosing spondylitis in the Taiwanese population
Source: J Transl Med. 2022 Dec 12;20:589. doi: 10.1186/s12967-022-03701-3 (PMC9746141; doi:10.1186/s12967-022-03701-3)
Supplement: Supplementary file 5 — Additional file 5: Figure S1. Comparison of AS polygenic risk between AS cases and controls. Figure S2. Strata plot with ten strata of increasing PRS versus prevalence (%) of developing AS. [file 12967_2022_3701_MOESM5_ESM.pdf]

Supplementary Figure

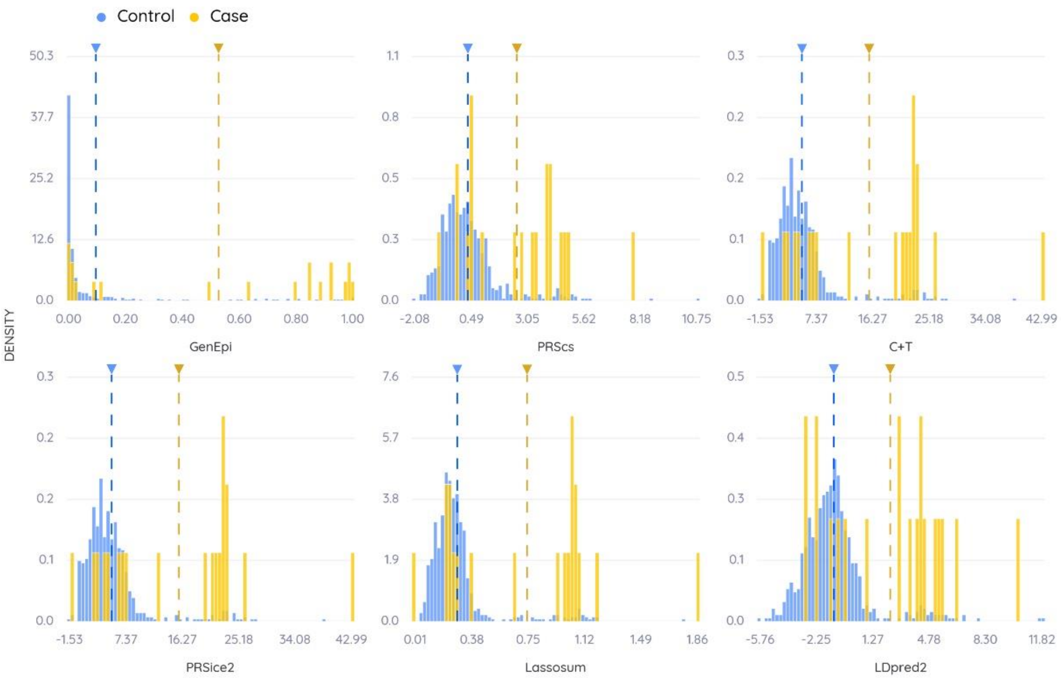

Supplementary Figure S1. **Comparison of AS polygenic risk between AS cases and controls.** The polygenic risk scores (PRS) were predicted by six methods. The blue dashed line with a blue triangle mark represents the median PRS of the control group, and the yellow dashed line with a yellow triangle mark represents the median PRS of the case group.

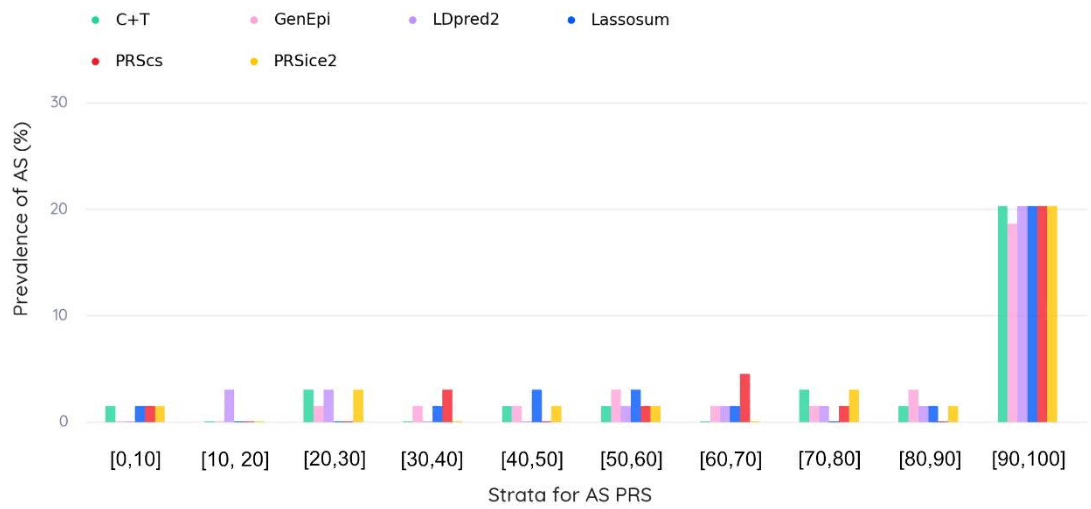

Supplementary Figure S2. **Strata plot with ten strata of increasing PRS versus prevalence (%) of developing AS.** The ten strata were ordinally ranked according to PRS prediction. The prevalence of AS was calculated as the percentage of AS patients in each stratum. The strata plot indicates that the top decile showed a striking increase of the prevalence percentage in the last stratum.
